# Supplementary material for: All-at-once RNA folding with 3D motif prediction framed by evolutionary information
Source: Nat Methods. 2025 Oct 3;22(10):2094–106. doi: 10.1038/s41592-025-02833-w (PMC12510878; doi:10.1038/s41592-025-02833-w)
Supplement: Supplementary file 3 — Source code to run CaCoFold-R3D. Data and results. [file 41592_2025_2833_MOESM3_ESM.gz › supplemental_material/Tables/Table_S1.pdf]

Type	Motif name	PDB	Rfam homology	CaCoFold-R3D	Other Methods	Motif Model
HL	GNRA-tetraloop	7EI6	synthetic	170	–	Hendrix *et al.*, 2005 [32]
HL	U-turn	4WFL	RF00017_Metazoan_SRP	YES	–	Kempf *et al.*, 2014 [37]
HL	UNCG-tetraloop	1F7Y	synthetic	48	–	Hendrix *et al.*, 2005 [32]
HL	ANYA-tetraloop	6MSF	synthetic	18	–	Hendrix *et al.*, 2005 [32]
HL	CUYG-tetraloop	2L6I	synthetic	20	–	Hendrix *et al.*, 2005 [32]
HL	YGNN-tetraloop	1AFX	synthetic	13	–	Hendrix *et al.*, 2005 [32]
HL	GANC-tetraloop	3BWP	synthetic	15	–	Keating *et al.*, 2009 [38]
HL	UNAC-tetraloop	4A4R	synthetic	26	–	Zhao *et al.*, 2012 [101]
HL	T-loop-tetraloop	7SAM	RF01085_TLS-PK4	YES	–	RMfam [27]
HL	L8_RNaseP_bact_a	1U9S	RF00010_RNaseP_bact_a	YES	–	Das & Baker, 2010 [16]
HL	Sarcin-Ricin_loop	1SCL	RF02540_LSU_rRNA_archaea	YES	BayesPairing2	Szewczak *et al.*, 1993 [87]
HL	CsrA_binding	2MF0	RF00018_CsrB	YES	–	Duss *et al.*, 2014 [20]
HL	GAGUA_pentaloop_sars	1XJR	RF00164_Coronavirus s2m motif	YES	–	Das & Baker, 2010 [16]
IL	Kink-turn_SAM_riboswitch	2GIS	RF00162_SAM_riboswitch	YES	BayesPairing2	Das & Baker, 2010 [16]
IL	Kink-turn_rRNA	1JJ2	RF02540_LSU_rRNA_archaeal	YES	RMDetect	Das & Baker, 2010 [16]
IL	LoopE	354D	RF00001_5S_rRNA	YES	–	Das & Baker, 2010 [16]
IL	GAAA_Tetraloop-receptor	1GID	RF00028_Group I intron	YES	–	Cate *et al.*, 1996 [7]
IL	C-loop	1S72	RF02541_LSU_rRNA_bacterial	YES	RMDetect	Lescoute *et al.*, 2005 [48]
IL	G-Bulge	3DIL	RF00168_Lysine riboswitch	YES	RMDetect	Serganov *et al.*, 2008 [82]
IL	G-Bulge_Das	1Q9A	RF02541_Bacterial LSU_RNA	YES	–	Das & Baker, 2010 [16]
IL	tandem GA	1SA9	RF00177_SSU_rRNA_bacteria	YES	RMDetect	Cruz & Westhof, 2011 [14]
IL	Twist up	1J5E	RF02541_LSU_rRNA_bacterial	NO, 9	–	Zhong & Zhang, 2011 [102]
IL	UAA/GAN	6AHR	RF00009_RNaseP_nuc	YES	–	Lee *et al.*, 2006 [43]
IL	J4a/4b	2QBZ	RF00380_M-box_riboswitch	YES	–	Das & Baker, 2010 [16]
IL	J4/5	2R8S	RF00028_Group I intron	NO, 6	–	Das & Baker, 2010 [16]
IL	GUA/GG_RRE	1CSL	RF00055_HIV-1 RRE	too conserved, 10	–	Das & Baker, 2010 [16]
IL	SRP RNA DOMAIN IV	1CQ5	RF00017_Metazoan_SRP	YES	–	Schmitz *et al.*, 1999 [81]
IL	Hook_turn_GAAC/CUA	1MHK	synthetic	32	–	Das & Baker, 2010 [16]
IL	Docking elbow-IL	3Q1Q	RF00011_RNaseP_bact_b	YES	–	Lehmann *et al.*, 2012 [44]
IL	pK-turn	3OK7	RF00010_RNase P RNA	NO, 45	–	Meyer *et al.*, 2012 [56]
J3	Purine_riboswitch_J3	2EEW	RF00167_Purine riboswitch	YES	–	Das & Baker, 2010 [16]
J3	hammerhead_CUGA/A/C	359D	RF02275_Hammerhead_HH9	YES	–	Das & Baker, 2010 [16]
J3	J3_typeA	4RZD	RF02680_PreQ1-III	YES	–	Lescoute & Westhoff, 2006 [49]
J3	J3_typeB	1J5E	RF00177_SSU_rRNA_bacteria	YES	–	Lescoute & Westhoff, 2006 [49]
J3	J3_typeC	4LX6	RF03165_2dG-II riboswitch	YES	–	Lescoute & Westhoff, 2006 [49]
J3	J3_TPP	2GDI	RF00059_TPP riboswitch	YES	–	Serganov *et al.*, 2006[83]
J3	J3_groupII	5J01	RF02001_group-II-D1D4-3	YES	–	this work
J4	HCV_IRES_AA/0/U/0	1KH6	RF00061_IRES_HCV	YES	–	Das & Baker, 2010 [16]
J4	J4_U1RNA	3PGW	RF00003_U1 RNA	YES	–	Weber *et al.*, 2010 [95]
BS	T-loop	1EHZ	RF00005_tRNA	YES	–	Hendrix *et al.*, 2005 [32]
BS	LoopE-a	4FRN	RF01689_AdoCbl-II	YES	–	this work
BS	LoopE-b	4FRN	RF01689_AdoCbl-II	YES	–	this work
BS	CRC_binding	-	RF00195_RsmY	YES	–	Sonnleitner *et al.*, 2009 [85]
PK		5DDP	RF01739_Glutamine riboswitch	YES	–	Ren *et al.*, 2015 [70]

Table S1. **Non-canonical motif *de novo* prediction from alignments.** For each motif, we identify one crystal structure that includes the motif, and we test for the prediction of the motif on the corresponding Rfam alignment. We also report other methods that have identified the motif in the same family. For motifs associated to a crystallized synthetic oligo, or not identified by CaCoFold-R3D, we provide the number of other Rfam families that include that motif with covariation support. The specifications of the motifs are provided in the R3D descriptor file given in Extended Figure S3. CaCoFold-R3D results for the Rfam seed aligments are given in the supplemental materials file `results/CaCoFold-R3D/CaCoFold-R3D_Rfam`.
